# The interventional value and research progress of vaginal microbiota transplantation in improving female sexual quality of life associated with dysbiosis of the reproductive microecosystem

**DOI:** 10.3389/fmed.2026.1812754

**Published:** 2026-04-13

**Authors:** Runzhi Shi, Xi Ye, Yangyang Tan, Ruxue Zhou, Xiangjie Zheng, Liehong Wang

**Affiliations:** 1 Clinical Medical College of Qinghai University, Xining, Qinghai, China; 2 Qinghai Red Cross Hospital, Xining, Qinghai, China

**Keywords:** female sexual quality of life, genital tract microbiota, sexual dysfunction, vaginal laxity, vaginal microbiota transplantation

## Abstract

Dysbiosis of the female genital tract microbiota represents a key pathological basis underlying clinical symptoms such as vaginal dryness and dyspareunia, which subsequently contribute to diminished sexual quality of life (QoL) in women. Although conventional antimicrobial therapies may provide short-term relief from infectious symptoms, they often fail to restore vaginal microbial homeostasis—thereby exhibiting inherent limitations, including high recurrence rates and increased risks of antimicrobial resistance. Vaginal microbiota transplantation (VMT), an emerging ecological restoration strategy, aims to reconstitute the recipient’s vaginal microbiota by transferring a complete, functionally intact microbial community from a rigorously screened healthy donor. This review systematically delineates the multidimensional mechanistic links between genital tract dysbiosis and impaired female sexual quality of life; elucidates the core clinical value of VMT; comprehensively summarizes recent advances in both preclinical research and clinical trials; and critically examines major challenges currently facing the field—including the lack of standardized donor screening criteria, variability in procedural protocols, insufficient long-term safety data, and evolving ethical and regulatory considerations. Furthermore, we propose forward-looking perspectives on future directions, such as the development of individualized, precision-based VMT approaches; the establishment of standardized, evidence-based technical frameworks; and the acceleration of clinical translation and implementation. This review is intended to provide a robust theoretical foundation and practical guidance for the scientifically sound promotion, standardized application, and further investigation of VMT—ultimately advancing women’s reproductive health and holistic sexual well-being.

## 1 Introduction

The ecological balance of the female genital tract is a cornerstone of reproductive health (1). The vaginal microbiome constitutes a dynamic, homeostatic system shaped collectively by the vaginal mucosa, resident microbial communities, endocrine regulatory networks, and the local immune system. Within this system, the vaginal microbiota serves as the central functional unit (2), engaging in bidirectional interactions with the host. Its composition is profoundly influenced by daily behavioral patterns and lifestyle factors, thereby directly impacting vaginal health (3). The vaginal microbiota plays an indispensable role in maintaining reproductive health, modulating local immune responses, and preserving sexual functional homeostasis. Dysbiosis—characterized by a reduction in beneficial, *Lactobacillus*-dominant commensals alongside aberrant proliferation of opportunistic anaerobes and yeast-like fungi—can precipitate common urogenital disorders, including bacterial vaginosis (BV), vulvovaginal candidiasis, and atrophic vaginitis (4). Such dysbiosis often manifests clinically as vaginal dryness and dyspareunia, leading to a marked decline in female sexual quality of life (QoL).

Current clinical management predominantly relies on symptomatic treatment with antibiotics or antifungal agents (5). Although these interventions provide short-term symptom relief, they often exacerbate microbial dysbiosis, impair host colonization resistance, and promote the emergence of antimicrobial resistance—key factors in the development of refractory vaginitis (6–9). In recent years, VMT has emerged as a novel, targeted strategy for ecological restoration. VMT involves the transfer of a carefully screened, intact vaginal microbial community from a healthy donor to a recipient, with the aim of restoring structural integrity, functional activity, and host–microbe interaction networks within the genital tract microbiome. This approach offers a promising, mechanism-based therapeutic avenue for sexual dysfunction arising from microbiota dysbiosis (10).

This review systematically examines the mechanisms linking VMT to improved quality of life (QoL) in women with vaginal dysbiosis, including VMT’s intervention mechanisms and clinical value, recent advances, donor screening criteria, and outcome assessment frameworks. Its core innovation lies in shifting focus from prior narrative and systematic reviews—which primarily addressed dysbiosis itself or associated inflammatory conditions—to “sexual QoL” as the central entry point. It explicitly integrates VMT’s ecological restoration effects with sexual function homeostasis, and theoretically highlights the underexplored host–microbe interaction network underlying sexual dysfunction.

Focusing on “sexual QoL” offers unique academic and clinical value: first, it centers patient needs, addressing a key gap in existing research—its predominant emphasis on disease symptoms over QoL improvement—and thereby broadens the scope of VMT’s clinical relevance; second, it provides novel therapeutic insights for dysbiosis-related sexual dysfunction, supporting VMT’s evolution from “disease treatment” to “QoL enhancement.” Ultimately, this perspective informs the standardization of VMT in clinical practice and guides future research.

## 2 Mechanisms underlying the association between genital microbiota dysbiosis and reduced female sexual quality of life

### 2.1 Disruption of the acidic microenvironment impairs the vaginal mucosal barrier function

In healthy women, the vaginal microbiota is dominated by *Lactobacillus* species, which maintain a local, mildly acidic microenvironment (pH 3.8–4.5) by secreting antimicrobial metabolites, including hydrogen peroxide, bacteriocins, and short-chain fatty acids. This acidic milieu not only effectively suppresses colonization by potential pathogenic microorganisms but also synergistically enhances the structural integrity and barrier function of the vaginal epithelium (11). Dysbiosis of the vaginal microbiota disrupts this acidic environment, thereby compromising mucosal barrier function. Emerging evidence suggests that impaired vaginal mucosal barrier function may serve as a central mediating mechanism linking vaginal disorders to sexual dysfunction (12); however, the quantitative relationship between the degree of mucosal barrier impairment and the severity of sexual dysfunction remains unclear and warrants further validation in larger-scale cohort studies. A hallmark of vaginal dysbiosis is a marked reduction in *Lactobacillus* abundance, leading to loss of acidic homeostasis. Concurrently, opportunistic anaerobic bacteria—such as *Gardnerella vaginalis* and *Atopobium vaginae*—and conditionally pathogenic fungi—such as *Candida albicans*—exhibit aberrant proliferation (13).

### 2.2 Symptoms arising from vaginal microbiota dysbiosis directly impair sexual experience

Bilardi et al. (14) reported that vaginal microbiota dysbiosis exerts its most pronounced negative impact on sexual functioning and self-esteem. Patients frequently avoid sexual activity—particularly oral sex—due to distressing symptoms such as malodor, abnormal vaginal discharge, and dyspareunia, often accompanied by feelings of shame and embarrassment. Consequently, sexual satisfaction and the quality of intimate relationships are significantly diminished. Giovannetti et al. (15) identified the hallmark symptoms of vaginal dysbiosis—including malodor, abnormal discharge, pruritus, and dyspareunia—and demonstrated that these manifestations contribute to inadequate vaginal lubrication, painful coital friction, impaired sexual arousal, and difficulty achieving orgasm. Scairati et al. (16) further noted that in postmenopausal women, dysbiosis is commonly characterized by a reduction in lactobacilli and a concomitant elevation in vaginal pH, leading to vaginal atrophy, dryness, dyspareunia, and disturbances in both sexual arousal and orgasmic function. Collectively, these symptoms may initiate and perpetuate a self-reinforcing vicious cycle—“symptom onset → avoidance of sexual activity → interpersonal strain”—thereby exerting multidimensional adverse effects on women’s physical health, psychological well-being, and relational intimacy.

### 2.3 Vaginal microecological imbalance–mediated immune dysregulation and psychological factors exacerbating sexual dysfunction

Dysbiosis of the genital tract microbiota can activate local immune–inflammatory responses, leading to significant upregulation of pro-inflammatory cytokines—including interleukin 1β (IL-1β) and tumor necrosis factor alpha (TNF-*α*)—and consequently inducing persistent vaginal mucosal inflammation (17). Thomas et al. (18) reported that vaginal microbial imbalance triggers chronic mucosal inflammation, characterized by sustained elevation of IL-1β, TNF-*α*, and IL-6, thereby disrupting both local and systemic immune homeostasis. This chronic inflammatory state, together with associated symptoms, contributes to heightened anxiety, depression, shame, and psychological stress—factors that further suppress immune function and exacerbate microbial dysbiosis, establishing a self-perpetuating vicious cycle. Moreover, the authors emphasized that psychological stress and negative affect directly impair sexual function by promoting avoidance of intercourse (due to anticipated or actual pain), blunting sexual arousal, and reducing sexual satisfaction—thereby indirectly worsening sexual dysfunction. Scairati et al. (16) demonstrated in a clinical study that vaginal dysbiosis is associated with immune suppression and concomitant psychological sequelae—including self-devaluation, anxiety, and sexual avoidance—which collectively contribute to the indirect aggravation of sexual dysfunction. Amabebe (19) further posited that chronic inflammation (driven by immune dysregulation) elevates cortisol levels, intensifying psychological stress, anxiety, and depressive symptoms; conversely, psychological stress reciprocally suppresses immune competence, forming a detrimental feedback loop involving the microbiota–immune–psychological axis. Bilardi (14) highlighted that patients with vaginal dysbiosis frequently exhibit active behavioral avoidance of sexual activity due to feelings of shame, anxiety, and fear of dyspareunia, resulting in markedly reduced sexual satisfaction. Accordingly, psychological factors serve as a critical indirect mediator of sexual dysfunction in this context. Additionally, emerging evidence indicates a positive association between the bacterial order *Gastranaerophilales* and indicators of psychological well-being (20), while psychological status exerts a sustained influence on sexual quality of life. Clinical data consistently show that the prevalence of anxiety and depression is significantly higher among patients with vaginitis than in healthy controls; furthermore, sexual quality-of-life scores demonstrate a statistically significant negative correlation with standardized psychological assessment scale scores (21, 22).

Collectively, these findings underscore that vaginal microbiota–mediated immune dysregulation and associated psychological factors constitute an essential pathophysiological component in the development and perpetuation of sexual dysfunction.

**Figure 1:**
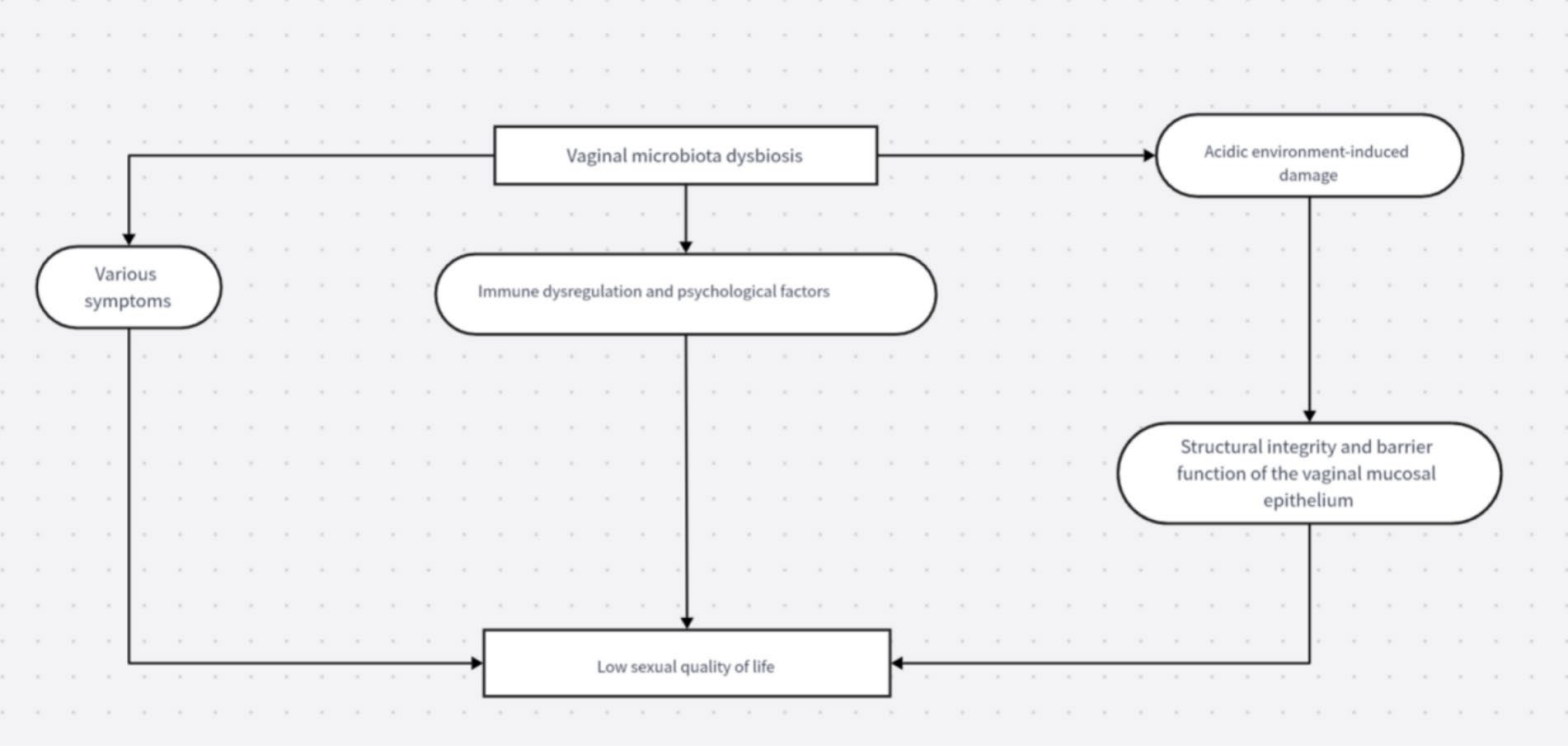
Mechanistic flowchart of vaginal microbiota dysbiosis and sexual dysfunction.

The association between vaginal microbiota dysbiosis and sexual dysfunction is illustrated in Figure 1.

## 3 Vaginal microbiota transplantation: interventional mechanisms and core value

### 3.1 Core interventional mechanism of VMT: restoration of a healthy vaginal microecological homeostasis

VMT draws upon the ecological modulation principles of fecal microbiota transplantation (FMT) (23). It involves the standardized preparation and subsequent transplantation of intact, rigorously screened vaginal microbial communities—sourced from healthy, reproductive-age female donors—into the recipient’s vagina to achieve precise reconstruction of the vaginal microbiota structure. Its core mechanisms of action encompass three interrelated aspects: First, rapid colonization by dominant commensal taxa and restoration of ecological niches. Following transplantation, *Lactobacillus* species rapidly adhere to and colonize the vaginal mucosal epithelium, occupying critical ecological niches. This reestablishes and sustains an acidic microenvironment with pH < 4.5, thereby effectively suppressing the adhesion, colonization, and proliferation of pathogenic microorganisms. Second, repair and reinforcement of the vaginal mucosal barrier function. A healthy microbial community enhances vaginal epithelial homeostasis by secreting metabolites—including short-chain fatty acids and hydrogen peroxide—which collectively promote epithelial cell proliferation and differentiation, upregulate tight junction protein expression (e.g., claudin-1, occludin, and zonula occludens-1 [ZO-1]), improve mucosal integrity, and reduce inflammatory cell infiltration. These effects collectively alleviate clinical symptoms such as vaginal dryness, burning sensation, and dyspareunia. Third, modulation of local immune homeostasis. The transplanted microbiota regulates innate immune responses within the vaginal mucosa, promoting the activation and expansion of regulatory T cells (Tregs) while downregulating the expression of pro-inflammatory cytokines, including TNF-*α* and IL-1β, thereby mitigating chronic low-grade inflammation. At the pathophysiological level, this intervention disrupts the causal link between vaginal dysbiosis and sexual dysfunction.

Compared with probiotic interventions based on single strains or limited bacterial consortia, VMT delivers a naturally derived, functionally integrated microbial community characterized by complex interspecies interactions and functional redundancy. This confers superior capacity to establish stable, resilient symbiotic relationships within the host vaginal niche, resulting in more durable and physiologically relevant microbiota reconstruction.

### 3.2 Core interventional value of VMT in enhancing sexual quality of life

(1) Alleviation of Clinical Symptoms and Restoration of Physiological Function: VMT significantly ameliorates core symptoms—including pruritus, burning pain, and abnormal vaginal discharge—in patients with recurrent vaginitis, while effectively reducing disease recurrence rates. A 2021 study by Kehong Wei et al. demonstrated the long-term therapeutic efficacy of VMT in recurrent bacterial vaginosis (BV) (24). Specifically, VMT achieved significantly higher symptom resolution rates and superior vaginal microbiota restoration compared with standard antibiotic regimens; notably, it facilitated stable reconstitution of a healthy vaginal microbiota dominated by *Lactobacillus* species.
(2) Improvement of Psychological Well-being and Restoration of Intimate Relationships: By providing sustained relief from physical symptoms and markedly lowering recurrence risk, VMT effectively mitigates anxiety and depressive symptoms associated with chronic disease burden, thereby enhancing patients’ self-identity and sexual self-confidence. Behaviors involving avoidance of sexual contact due to symptom-related distress decrease significantly; concurrently, both the frequency and satisfaction of intimate interactions between partners increase. Standardized psychometric assessments further confirm a statistically significant improvement in sexual self-confidence. Importantly, these psychological benefits and physiological recovery exhibit bidirectional positive modulation, jointly contributing to enhanced overall sexual health and quality of life in affected women.
(3) Overcoming Limitations of Conventional Therapy and Enabling Precision Intervention: Conventional antibiotic therapy frequently disrupts vaginal microbial community structure, contributing to high recurrence rates in BV and vulvovaginal candidiasis (VVC); multiple factors have been identified as positively correlated with recurrence (25). Moreover, antibiotics lack specificity for correcting the underlying dysbiosis—the fundamental pathogenic mechanism. In contrast, VMT represents a live-bacterial, functionally targeted microbiota-based intervention that directly addresses the core pathological feature of vaginal dysbiosis. It exerts dual therapeutic effects: reconstruction of a resilient, health-associated microbial community and restoration of colonization resistance. Critically, VMT circumvents risks associated with antimicrobial resistance and avoids secondary microbiota injury. As such, it offers an evidence-based alternative therapeutic strategy for patients with recurrent or refractory genitourinary microbiota disorders who have failed standard treatments—particularly for women whose persistently impaired sexual quality of life is attributable to microbiota dysbiosis.

## 4 Advances in vaginal microbiota transplantation

### 4.1 Progress in basic research: mechanistic insights and microbial community optimization

At the fundamental research level, scholars are progressively elucidating the molecular mechanisms underlying VMT (26) and systematically optimizing the compositional architecture of transplanted microbial communities. In recent years, rapid advances in molecular biology techniques have provided novel methodological tools and conceptual frameworks for vaginal microbiome research—particularly metagenomic approaches based on high-throughput sequencing—which have yielded breakthrough insights into the structural composition and functional gene repertoire of the vaginal microbiota, thereby inaugurating a new era in the investigation of associations between vaginal microbiota and human disease (25). Furthermore, the application of integrated multi-omics analyses—including metagenomics, metatranscriptomics, metabolomics, and host immunomics—has significantly deepened our understanding of the dynamic interactions between transplanted microbiota and the recipient’s vaginal mucosa. Such integrative approaches facilitate the precise identification of key molecular targets linking *Lactobacillus* engraftment, restoration of host immune homeostasis, and improvement of sexual function, thereby providing a robust theoretical foundation for the individualized and precision-oriented clinical implementation of VMT.

### 4.2 Clinical research advances: efficacy validation and therapeutic application expansion

Clinically, VMT-related research covers multiple types of clinical studies, including clinical trials, case series, and reviews, with distinct strengths and limitations that collectively shape the current evidence landscape. Below is a systematic classification, efficacy validation, and critical evaluation of these clinical studies, along with an analysis of existing evidence gaps, in line with reviewer requirements.

#### 4.2.1 Controlled clinical studies (including clinical trials and RCTs)

Controlled clinical studies—especially randomized controlled trials (RCTs)—represent the highest level of clinical evidence due to their rigorous design, control of confounding factors, and ability to minimize bias. Their primary strength lies in the reliable validation of therapeutic efficacy and safety through comparison with control groups, enabling objective evaluation of VMT’s superiority over conventional therapies. However, their limitations are equally prominent: the design is complex, implementation is difficult, and large-scale, multicenter RCTs remain scarce in the VMT field. In the latest clinical trial, Hussain et al. (27) reported that VMT can stably and durably restore a healthy vaginal microbiota—outperforming single-strain probiotics—and thus offers a novel, effective therapeutic strategy for recurrent BV. This study highlights the advantages of controlled clinical trials in confirming VMT’s efficacy, though it still requires validation in larger cohorts. Fatima A. Hussain et al. (27) further reported that VMT outperformed single-strain probiotic interventions in clinical outcomes and holds promise as a potential standard therapeutic modality for dysbiosis-related genital tract disorders—though this requires formal validation in larger, controlled trials. Scholars have emphasized that the next critical step is to conduct large-scale, multicenter RCTs to establish VMT as a first-line treatment (24), which is essential to address the current scarcity of high-quality control (QC) evidence.

#### 4.2.2 Observational clinical studies (case series and early clinical explorations)

Observational clinical studies, including case series and early clinical explorations, are the most common types of VMT research to date, with distinct strengths and limitations. Their primary advantage is their feasibility and convenience in initial efficacy exploration, especially for rare or complex conditions where controlled trials are difficult to implement, providing valuable preliminary clinical clues. However, their core limitations include small sample sizes, lack of control groups, and high susceptibility to bias, leading to limited reliability and generalizability of conclusions. Kumar’s article (28) noted that early clinical studies on VMT indicate that therapeutic outcomes vary substantially across individuals; host genetic factors, baseline vaginal microbial composition, immune status, and environmental influences may collectively modulate treatment efficacy—highlighting the necessity of personalized intervention strategies, but these findings are based on early observational data with insufficient bias control. Wrønding et al. (29) reported that antibiotic-free VMT can fully reverse severe vaginal dysbiosis; successful engraftment was directly associated with improved clinical pregnancy outcomes, offering a novel microbiota-based intervention strategy for recurrent pregnancy loss—this study provides important preliminary evidence but is limited by its observational design without a control group. A 2025-published case series demonstrated that VMT significantly alleviated coital pain and reduced libido in patients with complex genital tract infections, with no severe adverse events reported post-transplantation; while this study confirms VMT’s safety and potential efficacy in a specific population, its small sample size and lack of controls limit the generalizability of its conclusions.

#### 4.2.3 Clinical reviews and summary studies

Clinical reviews integrate existing clinical evidence to provide a comprehensive overview of VMT’s safety and efficacy, with the advantage of summarizing findings from multiple studies and identifying research gaps. In the latest VMT review (24), VMT has been consistently confirmed as safe and effective, with a significantly higher long-term remission rate compared to conventional therapies—this summary provides a holistic perspective on VMT’s clinical value but relies on the quality of existing primary studies. However, such reviews also have limitations: they cannot provide new empirical evidence and are prone to bias if the included studies are of low quality or poorly designed.

#### 4.2.4 Current clinical evidence gaps and future directions

Synthesizing the above clinical studies, the current VMT clinical evidence system has obvious gaps: high-quality, large-scale multicenter RCTs are scarce, and the majority of the studies are observational (case series or early clinical explorations) with low evidence levels; there is a lack of systematic correlation between different types of studies, failing to form a complete evidence chain; the efficacy of VMT varies across individuals, but personalized intervention strategies lack sufficient evidence support; robust empirical evidence remains scarce regarding whether microbiome-based interventions—particularly those containing viable beneficial bacteria—can reliably establish and sustain an optimal vaginal microbial community structure (30). Concurrently, ongoing clinical research aims to optimize VMT protocols—including donor microbiota processing methods, timing of transplantation, and dosing frequency—to improve engraftment efficiency and therapeutic consistency. Additionally, scholars have emphasized the necessity of enhancing public and professional awareness of the vaginal microbiota and VMT (27), as well as conducting more high-quality, standardized clinical studies to fill existing evidence gaps and expand VMT’s clinical applications beyond recurrent BV to recurrent vulvovaginal candidiasis (RVVC), mixed vaginitis, and associated sexual dysfunction.

## 5 Donor screening criteria, standard operating procedures, and key quality control measures, operational parameters, and quality assessment framework for vaginal microbiota transplantation

Donor screening and standardized operating procedures constitute the fundamental prerequisites for ensuring the safety and efficacy of VMT. Although a globally unified regulatory framework for VMT has yet to be established, current practices predominantly draw upon the well-established experience of fecal microbiota transplantation (FMT), while integrating the unique characteristics of the genital tract microbiome to develop a tailored technical system. Concurrently, clinical research continues to inform iterative refinement of procedural details. Flores-Treviño et al. (30) posit that donor selection remains a critical limiting factor in microbiota-based transplantation therapies. To address the time-intensive and costly nature of individual donor screening, the establishment of rigorously characterized fecal or vaginal microbial biobanks is recommended. Moreover, live biotherapeutic products (LBPs) derived from defined microbial consortia represent a promising therapeutic strategy—not only for recurrent Crohn’s disease but also for other disorders associated with dysbiosis. Such LBPs can be developed by isolating and functionally validating specific bacterial strains from qualified donors, thereby enabling targeted selection of beneficial microbes for precise disease indications. Personalized, microbiota-based therapeutic regimens further hold significant clinical promise as an effective approach to managing dysbiosis-related conditions. Below, we systematically outline evidence-based donor screening criteria and comprehensive procedural considerations for VMT, drawing on current literature and adapting key principles from established FMT guidelines (31–36). Moreover, this article will further explore the critical quality control procedures, operational parameters, and quality assessment framework for vaginal microbiota transplantation.

### 5.1 Donor screening criteria

**Figure 2:**
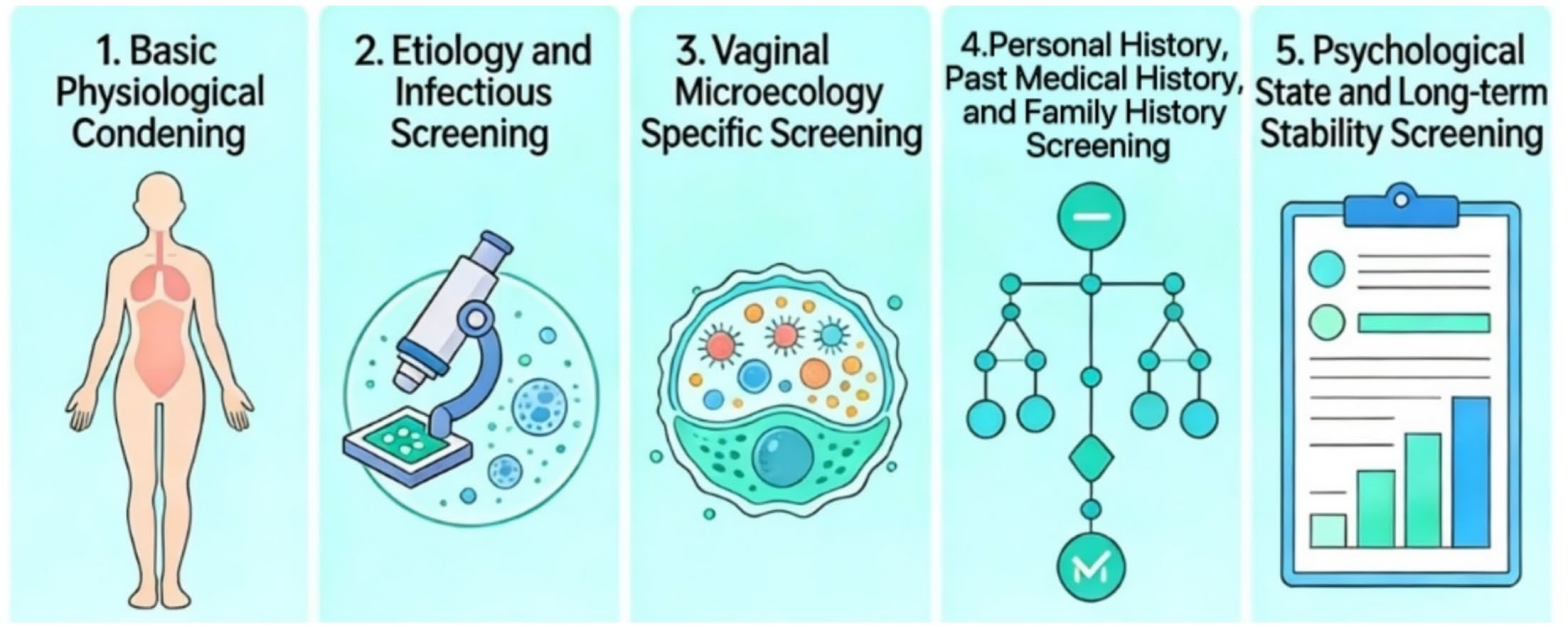
Donor screening criteria.

VMT donor screening is guided by the core principles of “health, stability, and safety,” requiring a multidimensional, systematic evaluation framework to rigorously exclude potential biological and clinical risks—thereby ensuring the high quality and non-pathogenicity of transplanted microbial communities. Specific screening criteria encompass the following five domains, as illustrated in Figure 2.

(1) Baseline Physiological Screening: Female donors aged 18–30 years—within their reproductive window—are prioritized. Body mass index (BMI) must fall strictly within the normal range of 18.5–23.9 kg/m^2^. This demographic typically exhibits high vaginal microbiota stability, dominant *Lactobacillus* relative abundance, and optimal overall physiological function. Comprehensive baseline hematological and biochemical assessments—including complete blood count, hepatic and renal function tests, serum electrolyte levels, and C-reactive protein—are mandatory to rule out underlying organic disease. Pregnancy and menstruation are explicitly excluded as eligibility criteria to mitigate confounding effects of cyclical estrogenic and progestogenic fluctuations on vaginal microbial composition.
(2) Pathogen and Infectious Disease Screening: As a critical barrier against iatrogenic infection, this tiered screening entails comprehensive pathogen detection across multiple specimen types. Blood samples must test negative for antibodies against hepatitis B virus (HBV), hepatitis C virus (HCV), human immunodeficiency virus (HIV), *Treponema pallidum*, Epstein–Barr virus (EBV), cytomegalovirus (CMV), and Severe Acute Respiratory Syndrome Coronavirus 2 (SARS-CoV-2). Vaginal secretions and other relevant specimens must be screened for common bacterial, fungal, parasitic, and viral pathogens—including *Clostridioides difficile*, *Campylobacter jejuni*, Salmonella spp., Shigella spp., *G. vaginalis*, and *C. albicans*—as well as parasitic ova/cysts (e.g., *Entamoeba histolytica* and *Trichomonas vaginalis*) and enteric/reproductive tract–associated viruses (e.g., norovirus, rotavirus). All results must be negative. Furthermore, molecular methods—including multiplex PCR and shotgun metagenomic sequencing—are required to detect key antimicrobial resistance genes (e.g., extended-spectrum *β*-lactamases, carbapenemases, and vancomycin resistance genes) to prevent the transmission of multidrug-resistant organisms.
(3) Vaginal Microbiota–Specific Screening: Donors must exhibit an “optimal vaginal microbiota” phenotype characterized by *Lactobacillus* dominance. Specifically, beneficial species—including *Lactobacillus crispatus* and *Lactiplantibacillus plantarum*—must demonstrate significantly higher relative abundance compared with other commensal taxa; conversely, anaerobic bacteria, fungi, and other potentially pathogenic or dysbiosis-associated taxa must be present at minimal levels. Vaginal pH must remain stably within the physiologically acidic range of 3.8–4.5.
(4) Personal, Medical, and Family History Screening: Information is systematically collected via standardized clinical interviews and structured questionnaires. Donors must confirm absence of gastrointestinal or lower genital tract symptoms within the preceding 2 weeks; no use of antibiotics, proton–pump inhibitors, H₂-receptor antagonists, immunosuppressants, probiotics, or other agents known to perturb microbial homeostasis within the prior 3 months substantially; and no history of blood transfusion, gastrointestinal surgery, or documented exposure to infectious diseases. Lifestyle assessment requires evidence of regular sleep–wake cycles and balanced nutrition, with explicit exclusion of smoking, alcohol consumption, substance use, or unprotected sexual activity. Donors must also report no skin or mucosal breaks within the past 6 months, no receipt of live attenuated vaccines, and no participation in any interventional drug clinical trials during that period. Family history is meticulously reviewed, with particular attention to first-degree relatives’ diagnoses of chronic gastrointestinal inflammatory disorders (e.g., inflammatory bowel disease), gastrointestinal malignancies, active infectious diseases, or monogenic hereditary conditions—including familial adenomatous polyposis (FAP) and Lynch syndrome (hereditary nonpolyposis colorectal cancer)—to mitigate risks associated with genetic susceptibility and comorbid disease predisposition.
Psychological Assessment and Longitudinal Stability Screening: A structured psychiatric or clinical psychological interview—supplemented by validated standardized instruments—is conducted, including the Self-Rating Depression Scale (SDQ), Self-Rating Anxiety Scale (SAS), and Pittsburgh Sleep Quality Index (PSQI), to ensure absence of clinically significant depressive, anxiety, or sleep disorders. Donors must provide a formal commitment to sustained, regular donation for a minimum duration of 6 months. Comprehensive re-evaluation—including all aforementioned screening parameters—is mandated every 2 months. Additionally, all donated microbial samples must be archived with duplicate aliquots, and each batch undergoes either 16S ribosomal RNA (rRNA) gene amplicon sequencing or whole-metagenome shotgun sequencing. This longitudinal monitoring strategy minimizes inter- and intra-individual microbiota–driven heterogeneity in therapeutic outcomes.

### 5.2 Operational procedure

**Figure 3:**
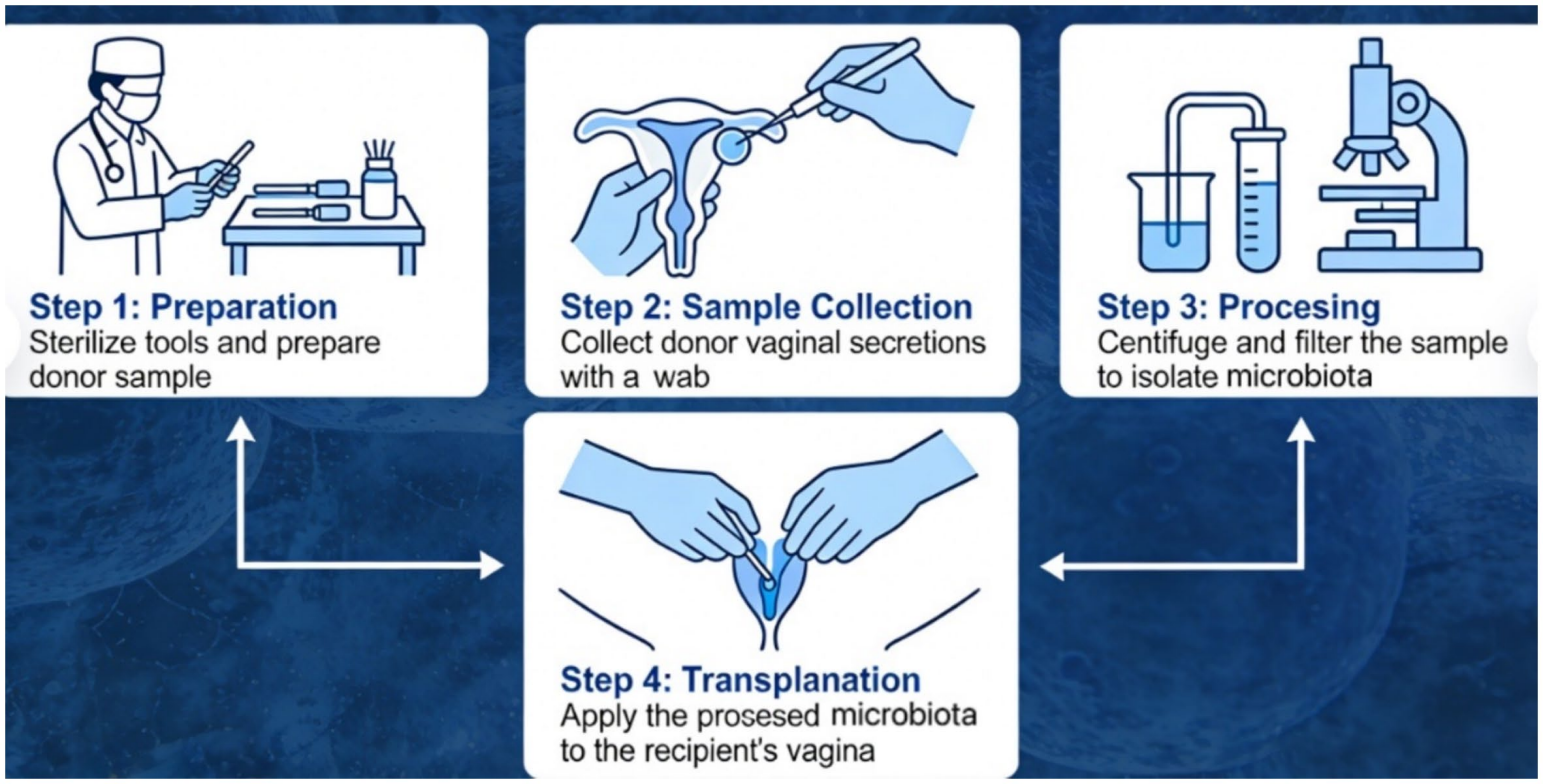
Operational procedure.

VMT must be performed strictly in accordance with aseptic techniques and standardized procedural protocols. The entire workflow comprises four core phases—donor sample collection, microbiota processing and preparation, quality control and traceability, and transplantation implementation—each designed to ensure microbial viability, biological safety, and full traceability throughout the process (Figure 3).

(1) Sample Collection: Collection is conducted in a dedicated, sterile sampling area compliant with the Technical Specifications for Disinfection in Healthcare Institutions. Donors must use sterilization-validated, single-use sterile containers specifically designed for vaginal secretion collection. The collected volume shall meet both downstream processing and clinical application requirements (recommended ≥5 mL). Secretions must exhibit a homogeneous, viscous, non-aqueous consistency—morphologically analogous to Bristol Stool Scale grades 3–5, reflecting stable physical integrity. Collection must be performed under strict conditions to prevent contamination by urine, water, or other exogenous sources. Immediately upon completion, samples are sealed and stored at 2–8 °C under cold-chain conditions or directly transported to the laboratory for immediate processing; the interval between collection and initiation of processing must not exceed 2 h to preserve microbial community structure and metabolic activity.
(2) Microbiota Processing and Preparation: All procedures are carried out in a clean laboratory certified for Biosafety Level 2 (BSL-2) containment. The laboratory must be functionally zoned into distinct areas: sample reception, cleaning and sterilization, microbial suspension preparation, and ultra-low-temperature cryopreservation. Unidirectional material transfer between zones is enforced via double-door interlocked pass-through chambers to eliminate cross-contamination risks. Freshly collected secretions are diluted 1:3 (v/v) with sterile saline under strict anaerobic conditions and thoroughly homogenized using vortex mixing. Subsequently, epithelial cells, mucus aggregates, and non-viable debris are removed via differential centrifugation and filtration. Anaerobic conditions are rigorously maintained throughout all steps to prevent inactivation of obligate anaerobes. For oral or intravaginal capsule formulations, lyophilization must follow a validated, programmable freezing protocol that incorporates a certified cryoprotectant system (e.g., a trehalose–mannitol composite). Final capsules undergo quantitative viability assessment: bacterial survival rate must be ≥85%, and viable cell density must be ≥10^9^ colony-forming units per gram (CFU/g), as determined by anaerobic plate culture.
(3) Quality Control and Traceability: Each production batch undergoes comprehensive quality testing, including but not limited to: (i) retesting for pathogenic microorganisms—including *Neisseria gonorrhoeae*, *Chlamydia trachomatis*, *Ureaplasma urealyticum*, herpes simplex virus (HSV), and high-risk human papillomavirus (HPV) genotypes; (ii) screening for antimicrobial resistance genes; (iii) quantitative aerobic and anaerobic CFU enumeration; and (iv) 16S rRNA gene amplicon sequencing to assess alpha- and beta-diversity metrics and relative abundances of core bacterial genera (e.g., *Lactobacillus*, *Gardnerella*, and *Prevotella*). Each batch is individually packaged in sterile, tamper-evident containers bearing complete labeling: donor unique identifier, collection date, preparation date, batch number, and expiration date. A minimum of one original aliquot of the microbial suspension or finished capsules must be retained for ≥6 months under appropriate storage conditions. Furthermore, all final products undergo a mandatory static observation period; upon its conclusion, repeat microbiological and biochemical assays—including nucleic acid testing for HIV, HBV, and HCV—are performed to mitigate the risk of missing pathogens during the serological “window period.” Only batches passing all release criteria are authorized for clinical use.
(4) Transplantation Implementation and Post-Procedure Follow-up: Prior to transplantation, recipients undergo systematic evaluation of baseline vaginal microecology—including pH measurement, microscopic examination for clue cells, and *lactobacillary* grade scoring. When indicated, gentle pre-transplant preparation (e.g., isotonic saline irrigation) may be performed to reduce local inflammation or suppress dominant pathogenic flora without compromising mucosal integrity. Intravaginal administration is the preferred route: the microbial suspension or sustained-release formulation (e.g., thermosensitive hydrogel or microsphere-based delivery systems) is slowly instilled into the posterior vaginal fornix to maximize contact with the vaginal epithelium and prolong residence time. Recipients are monitored clinically for ≥24 h post-transplantation, with structured documentation of mild adverse events—including pruritus, burning sensation, and alterations in discharge characteristics. A standardized adverse event grading and reporting framework—aligned with Common Terminology Criteria for Adverse Events (CTCAE)—is implemented to ensure consistent surveillance and timely intervention.

### 5.3 Key quality control measures, operational parameters, and quality assessment system for vaginal microbiota transplantation

#### 5.3.1 Key quality control measures for vaginal microbiota transplantation

Quality control (QC) is integrated throughout the entire VMT process, with particular emphasis on strengthening three core QC components to ensure the safety and efficacy of transplantation, prevent adverse events, and maintain the stability of therapeutic outcomes.

(1) Donor Microbiota QC: The core focus is the assessment of donor microbiota viability, purity, and safety—specifically confirming the absence of pathogenic contaminants, verifying that *Lactobacillus* viability meets predefined standards, and rigorously excluding potential microbial contaminants. Laboratories must strictly adhere to aseptic techniques; utilize sterile consumables and molecular-grade reagents; include sterile water as a negative control in all assays; and actively participate in external quality assessment (EQA) programs to ensure analytical reliability and result accuracy.
(2) Procedural QC: Aseptic technique must be rigorously maintained at every stage—from donor sample collection and processing to final transplantation—to eliminate contamination risks. Standardized operational parameters—including centrifugation speed, cryopreservation temperature, and administered transplant dose—must be strictly defined and consistently applied to ensure procedural reproducibility and compliance. Personnel involved in handling or administering the microbiota must wear full sterile attire (gowns, gloves, and masks) to prevent contamination from skin flora or respiratory droplets, thereby preserving microbiota viability and safeguarding procedural integrity.
(3) Post-Transplantation QC: Close clinical monitoring of recipients is required for early detection of adverse reactions—including vaginal bleeding, severe pruritus, or worsening infection—and a robust adverse event reporting and emergency response protocol must be established and implemented promptly to ensure patient safety. Concurrently, longitudinal monitoring of both donor and recipient vaginal microbiota composition is essential; intervention strategies should be dynamically adjusted based on observed microbial shifts to optimize therapeutic durability and minimize disease recurrence.

#### 5.3.2 Core operational parameters for vaginal microbiota transplantation

VMT operational procedures encompass donor microbiota collection, processing, preservation, transplantation, and postoperative care. Standardized parameters must be clearly defined for each step to ensure procedural consistency and optimal microbial viability.

(1) Donor Microbiota Collection Parameters: Collection should be performed 3–7 days after the cessation of menstruation, provided that the donor has abstained from sexual intercourse and vaginal medication or douching for at least 48 h. The collection must avoid the menstrual and ovulation periods. Aseptic technique is mandatory: the external genitalia and cervical os are disinfected prior to collection, followed by sampling of vaginal secretions using sterile collection tubes or specialized instruments. The collected volume should be strictly controlled at 5–10 mL—sufficient to meet transplantation requirements. Immediately after collection, specimens must be transferred into dedicated DNA stabilization or transport media and stored under refrigerated conditions (2–8 °C). Specimens must be delivered to the laboratory or processed within 24 h to prevent a decline in microbial viability.
(2) Microbiota Processing and Preservation Parameters: All processing steps must be conducted in a sterile environment, such as a laminar flow hood. Vaginal microbiota is purified via centrifugation-based separation to remove cellular debris, mucus, and non-microbial contaminants while preserving viable bacterial populations. When indicated, beneficial strains—such as *L. crispatus*—may be selectively enriched and co-cultured. Preservation strategies are stratified by duration: short-term storage is performed at 4 °C for up to 72 h; long-term storage utilizes ultra-low temperature freezing at −80 °C without cryoprotectants (e.g., glycerol), maintaining *lactobacilli* viability for over 6 months. Thawing must be performed gradually to minimize thermal shock and preserve microbial integrity.
(3) Recipient Preconditioning and Transplantation Parameters: Prior to transplantation, recipients undergo vaginal cleansing to eliminate abnormal secretions and pathogenic microorganisms. If antibiotic preconditioning is required, narrow-spectrum agents are preferred, with strict adherence to dosing regimens and treatment duration to avoid indiscriminate use of broad-spectrum antibiotics—which may compromise residual beneficial flora. During transplantation, processed microbiota suspension is delivered precisely to the vaginal fornix using a sterile catheter. Following administration, recipients are instructed to remain supine for 30–60 min to minimize efflux. For 72 h post-transplantation, sexual activity, vaginal medications, and douching are strictly prohibited to optimize engraftment and colonization.
(4) Postoperative Care Parameters: A standardized follow-up schedule is implemented, with clinical and microbiological assessments scheduled at 1 week, 2 weeks, 1 month, and 3 months post-transplantation to monitor symptom resolution and vaginal microbiome restoration. Recipients receive evidence-based counseling on hygiene practices—including avoidance of unnecessary antibiotics and excessive vaginal cleansing—as well as dietary recommendations (e.g., low-sugar, high-fiber intake) and lifestyle modifications aimed at enhancing systemic immunity and sustaining long-term vaginal eubiosis.

#### 5.3.3 Quality assessment system for vaginal microbiota transplantation

Establish a comprehensive quality evaluation system encompassing three core dimensions—therapeutic efficacy, safety, and vaginal microbiota—to objectively assess the intervention effects and safety profile of VMT, thereby providing robust evidence for clinical optimization.

(1) Efficacy Assessment: Primary evaluation centers on improvement in sexual quality of life, quantified using standardized, validated instruments such as the Female Sexual Function Index (FSFI). Specific endpoints include changes in dyspareunia, sexual satisfaction, and sexual self-confidence. Complementary assessment incorporates resolution of somatic symptoms—including vaginal pruritus, burning sensation, and abnormal vaginal discharge—to holistically determine therapeutic response. A minimum follow-up duration of 6 months is mandated to rigorously evaluate long-term efficacy and recurrence rates, ensuring reliable assessment of intervention durability.
(2) Safety Assessment: Systematic monitoring of adverse events is conducted throughout the post-transplantation period, with particular attention to infection dissemination, hypersensitivity reactions, and iatrogenic vaginal trauma—thereby evaluating short-term impacts of donor-derived microbiota on both systemic and genitourinary health. Long-term surveillance further addresses potential safety concerns, including microbial dysbiosis rebound and horizontal transfer of antimicrobial resistance genes. Proactive identification and timely management of emerging safety signals are integral to safeguarding patients’ long-term well-being.
(3) Microbiota Assessment: High-resolution profiling of the vaginal microbiota—using 16S rRNA gene sequencing—is performed pre- and post-VMT to quantify compositional shifts, *α*-diversity, *β*-diversity, relative abundance of dominant commensal taxa (e.g., *Lactobacillus* spp.), and enrichment of pathogenic or dysbiosis-associated microbes. This enables objective evaluation of vaginal microbial ecosystem restoration. Concurrently, a validated Microbial Balance Score is applied to stratify individual risk for recurrent BV and sexually transmitted infections (STIs), furnishing microbiologically grounded, quantitative evidence to inform both efficacy interpretation and personalized protocol refinement.

## 6 Discussion

VMT is an emerging biomedical intervention designed to restore a dysbiotic vaginal microbiome in recipients by transferring microbiota derived from healthy donors. This approach has demonstrated promising therapeutic potential in the management of refractory bacterial vaginosis, recurrent pregnancy loss, and other related gynecological disorders. However, VMT remains in the early stages of research and development: standardized operating procedures—encompassing critical aspects such as donor screening, sample preparation, administration routes, and quality control—have yet to be established. Consequently, its clinical translation and real-world implementation are inevitably accompanied by multifaceted safety risks, ethical challenges, and regulatory complexities. Moreover, robust evidence generation and interdisciplinary integration—including microbiology, reproductive immunology, bioinformatics, and regulatory science—are essential to broaden the analytical perspective and strengthen the scientific foundation of this modality. The following section will conduct an in-depth examination of the aforementioned core issues, existing challenges, and future prospects.

### 6.1 Potential security risks associated with VMT technology

VMT employs live microorganisms as its core therapeutic intervention, and safety risks permeate the entire procedural workflow. Among these, the most salient concern is the paucity of long-term follow-up data. To date, the majority of the clinical studies on VMT adopt small-sample, short-term designs with follow-up durations of less than 5 years, thereby lacking robust longitudinal evidence to comprehensively evaluate long-term safety. The long-term engraftment dynamics and evolutionary trajectories of transplanted microbial communities remain poorly characterized; such uncertainties raise concerns that recipient-specific physiological or immunological changes may trigger dysbiosis. Moreover, undetected opportunistic pathogens may persist within donor-derived inocula, yet their potential long-term impacts—particularly on reproductive health—remain empirically unverified. Consequently, establishing a scientifically grounded risk surveillance and early-warning framework remains challenging. Inadequate donor screening poses additional safety hazards: it increases the risk of transmitting rare pathogens or antimicrobial-resistant strains, and may also result in suboptimal microbial compatibility between donor and recipient—potentially precipitating recipient microbiota disruption. Furthermore, intrinsic donor characteristics—including age, hormonal status, microbiome composition, and health history—can significantly influence both the efficacy and safety profile of VMT. Beyond donor-related factors, non-standardized sample preparation and administration procedures may introduce contamination, compromise microbial viability, or cause mechanical injury to the vaginal mucosa. Inter-individual variability in host response further complicates safety assessment: immunocompromised individuals, patients with underlying comorbidities, and pregnant women represent particularly vulnerable populations, exhibiting heightened susceptibility to adverse events associated with VMT.

### 6.2 Ethical challenges facing VMT technology

VMT involves the rights of donors and recipients, as well as end-to-end management of biological samples; ethical challenges center on the uniqueness of informed consent and controversies in biological sample governance. Informed consent—the core ethical principle—is difficult to fully implement in VMT due to technological uncertainty: clinicians cannot comprehensively disclose risks and benefits, must balance donor and recipient preferences, and face added complexity with vulnerable populations—donors may not grasp potential impacts or privacy risks, while recipients—burdened by illness—may overlook risks. Ethical disputes over biological sample management stem primarily from ambiguous ownership and usage rights, leading to unauthorized or commercial use without donor consent; lack of standardized storage and usage protocols invites ethical conflict; and poor handling of sensitive data risks privacy breaches, harming donor and recipient rights. Additionally, VMT raises concerns about inequitable resource allocation, nonstandard donor recruitment, and unclear boundaries for clinical application—potentially exacerbating health inequities or enabling misuse.

### 6.3 Regulatory requirements for VMT technology and cross-national variations in regulatory pathways

The standardized development of VMT relies on a robust regulatory framework. Regulatory approaches vary significantly across countries due to differences in technological maturity and healthcare systems. No country has yet enacted dedicated VMT regulations; instead, oversight relies on general rules for biomedical innovations and biospecimen management—resulting in fragmented regulation. While clinical studies require review and registration, challenges persist, including inconsistent standards, insufficient enforcement, and unclear long-term follow-up requirements. The United States regulates VMT as a biologic product under strict approval; the European Union (EU) classifies it as an advanced therapy medicinal product (ATMP) under unified oversight; and Japan governs it as regenerative medicine. All three emphasize end-to-end regulation, ethical review, and long-term follow-up. Key regulatory differences lie in classification, procedural pathways, long-term follow-up mandates, and ethical oversight—rooted primarily in disparities in technological advancement, in the emphasis on individual rights protection, and in regulatory system maturity.

### 6.4 Evidence-based evidence

VMT represents a promising therapeutic strategy for restoring vaginal microbial homeostasis and ameliorating associated reproductive health and sexual function disorders. Although preliminary evidence—particularly from case reports and small-scale studies—suggests potential efficacy in refractory conditions such as recurrent BV, the current body of high-quality, reproducible evidence supporting its routine clinical adoption remains limited.

Existing research on VMT falls broadly into three categories: (i) case reports and small case series; (ii) controlled interventional studies; and (iii) narrative or systematic reviews and mechanistic investigations. Collectively, these studies suffer from substantial methodological limitations—including small sample sizes, high risk of bias, limited generalizability, and a paucity of long-term data on both efficacy and safety.

Therefore, robust evidence generation is urgently needed. Future research priorities should include large-scale, multicenter, randomized controlled trials (RCTs) with extended follow-up periods; standardized protocols for donor screening and VMT intervention; broader inclusion of diverse patient populations; and closer integration of mechanistic insights with clinical outcomes—thereby advancing the scientific rigor, standardization, and responsible translation of VMT into clinical practice.

### 6.5 Interdisciplinary field

From an interdisciplinary perspective, the deep integration of microbiome science and gynecology has opened a novel research dimension for elucidating the association between female reproductive health and sexual quality of life. Moreover, emerging evidence linking gut and genital tract dysbiosis to endometriosis has significantly expanded both the clinical applicability and theoretical depth of VMT.

Endometriosis—a highly prevalent and clinically complex gynecological disorder—is characterized by symptoms including dysmenorrhea, chronic pelvic pain, and infertility. Critically, it also exerts profound negative effects on pain perception, sexual function, and overall quality of life. Accumulating evidence from recent studies indicates that dysbiosis in both the gut and genital tract is closely associated with the pathogenesis and progression of endometriosis, establishing this interface as a pivotal focus within interdisciplinary microbiome–gynecology research. Systematic reviews and meta-analyses have demonstrated that patients with endometriosis exhibit significantly altered gut microbial diversity compared with healthy controls: Shannon and Simpson indices consistently reveal compositional disturbances in the gut microbiota. These alterations are thought to influence genital tract microbial homeostasis via the “gut–reproductive axis” (37). Concurrently, pronounced dysbiosis is observed in the genital tract microbiota of endometriosis patients—specifically, reduced Lactobacillus abundance and excessive proliferation of anaerobic bacteria. This dual-site dysbiosis not only exacerbates inflammatory responses and pain symptoms in endometriosis but also compromises vaginal mucosal barrier integrity, thereby further contributing to impaired sexual quality of life (38). As highlighted in the literature (39), precise modulation of the genital tract microbiome is a key strategy for improving quality of life in patients with gynecological disorders—providing robust scientific support for the clinical application of VMT.

Building upon these interdisciplinary advances, the therapeutic value of VMT in addressing sexual quality-of-life impairment linked to genital tract dysbiosis extends well beyond simple restoration of vaginal microbial ecology. Rather, VMT holds promise for synergistic intervention in endometriosis and related gynecological conditions through multi-target mechanisms—including modulation of the gut–reproductive axis, attenuation of chronic inflammation, and improvement of hormonal metabolism. Consequently, VMT contributes to the holistic enhancement of female sexual quality of life across multiple physiological dimensions—a paradigm that aligns with the emerging trend in microbiome research toward “multi-site coordinated regulation.”

### 6.6 Current challenges and future perspectives

Although VMT holds significant therapeutic promise, its clinical application faces four key challenges. First, donor screening and safety are insufficient: VMT carries risks of pathogen transmission, necessitating comprehensive testing for infectious, microbial, and metabolic diseases—and mandatory biobank traceability—yet no international screening standards exist. Second, long-term safety and durable efficacy remain unproven: current studies have short follow-up periods; large-scale, prospective, longitudinal cohort studies are urgently needed to assess engraftment stability, functional persistence, delayed adverse effects, and long-term impacts on pregnancy and reproductive health. Third, procedures lack standardization: no consensus guidelines govern critical steps—including donor sample collection, microbial processing, cryopreservation, formulation, and administration—leading to high methodological variability, poor reproducibility, and cross-study comparability. Fourth, ethical and perceptual barriers persist: patient acceptance of microbial interventions is generally low; clearer ethical consensus and regulatory guidance are needed on donor eligibility, informed consent, data privacy, and equitable access.

Future VMT research will center on three interlinked pillars: standardization, personalization, and clinical translation. For standardization, consensus-based frameworks are urgently required to unify donor screening, microbial preparation, product formulation, and clinical delivery—ensuring well-defined, rigorously quality-controlled, consistently safe, and reproducibly effective VMT therapies. For personalization, multi-omics integration—host genomics, baseline vaginal microbiota, metabolomics, and clinical phenotypes—will enable evidence-based donor matching or *de novo* design of synthetic consortia, enhancing engraftment and treatment precision. For clinical translation, large, multicenter, randomized controlled trials with extended follow-up are essential to define efficacy, optimal dosing, and target populations across indications. Concurrently, synergistic strategies—combining VMT with next-generation probiotics, prebiotics, or drugs—should be systematically explored to broaden clinical utility. Moreover, public and professional education must be strengthened, and ethical governance improved—including transparent informed consent, robust privacy protection, and adaptive regulation—to build trust and ensure responsible implementation. As evidence accumulates, VMT may restore vaginal microbial balance, significantly improve sexual and reproductive health, and extend into disease prevention—such as reducing cervical intraepithelial neoplasia risk and supporting gynecologic oncology.
