# Correction: The interventional value and research progress of vaginal microbiota transplantation in improving female sexual quality of life associated with dysbiosis of the reproductive microecosystem

**DOI:** 10.3389/fmed.2026.1858640

**Published:** 2026-05-05

**Authors:** Runzhi Shi, Xi Ye, Yangyang Tan, Ruxue Zhou, Xiangjie Zheng, Liehong Wang

**Affiliations:** 1 Clinical Medical College of Qinghai University, Xining, Qinghai, China; 2 Qinghai Red Cross Hospital, Xining, Qinghai, China

**Keywords:** female sexual quality of life, genital tract microbiota, sexual dysfunction, vaginal laxity, vaginal microbiota transplantation

Affiliation “Qinghai Red Cross Hospital, Xining, Qinghai, China” was omitted for author Liehong Wang. This affiliation has now been added for author Liehong Wang.

The original version of this article has been updated.

